# Experimental study and numerical analysis on axial compression of round-ended concrete filled CFRP-aluminum tube columns

**DOI:** 10.1371/journal.pone.0296005

**Published:** 2023-12-21

**Authors:** Mengjun Wang, Congrong Tang, Qirong Qiu, Yong Yu

**Affiliations:** 1 Nanjing Vocational Institute of Railway Technology, Nanjing, China; 2 Jiangsu Xilinghui Construction Engineering Co., Ltd, Nanjing, China; 3 Shanghai Construction Engineering Fifth Construction Group Co., Ltd. Shanghai, China; 4 School of Civil Engineering and Engineering Management, Guangzhou Maritime University, Guangzhou, China; 5 School of Environment and Civil Engineering, Dongguan University of Technology, Dongguan, China; University of Duhok, IRAQ

## Abstract

To enhance the concrete confinement ability of circular-ended aluminum alloy tubes, carbon fiber reinforced polymer (CFRP) was bonded onto the tube surface to form CFRP confined concrete columns with circular ends (RCFCAT). Eight specimens were designed with number of CFRP layers and section aspect ratio as variables. Axial loading test and finite element analysis were carried out. Results showed CFRP delayed buckling of the aluminum alloy tube flat surfaces, transforming inclined shear buckling failure into CFRP fracture failure. Specimens with aspect ratio above 4 experienced instability failures. Under same cross-section, CFRP increased axial compression bearing capacity and ductility by up to 30.8% and 43.4% respectively. As aspect ratio increased, enhancement coefficients of bearing capacity and ductility gradually decreased, the aspect ratio is restrictive when it is less than 2.5. CFRP strengthening increased initial axial compression stiffness of specimens by up to 117.9%. The stiffness decreased gradually with increasing aspect ratio, with most significant increase at aspect ratio of 4. Strain analysis showed CFRP bonding remarkably reduced circumferential and longitudinal strains. Confinement effect was optimal at aspect ratio around 2.0. The rationality of the refined FE model established has been verified in terms of load displacement curves, capturing circular aluminum tube oblique shear buckling, concrete "V" shaped crushing, and CFRP tearing during specimen failure. The parameter analysis showed that increasing the number of CFRP layers is one of the most effective methods for improving the ultimate bearing capacity of RCFCAT.

## Introduction

**Fig 1:**
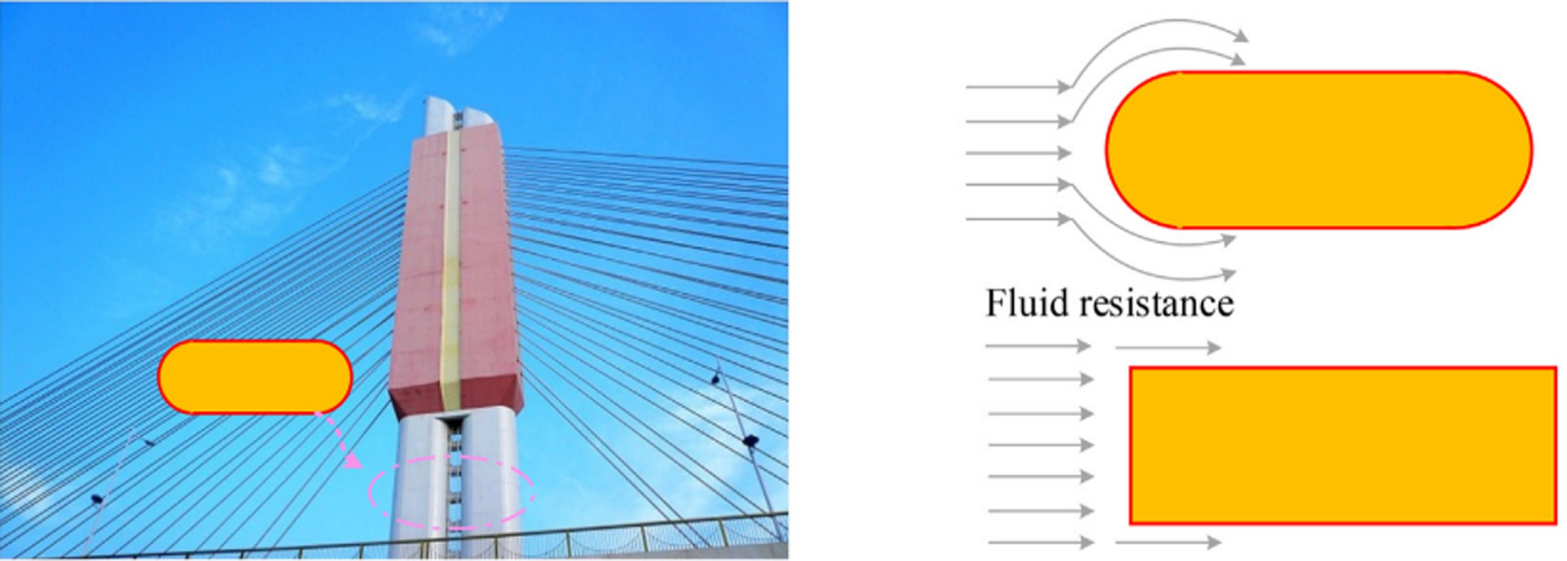
Actual engineering and cross-sectional characteristics of using RE-CFST columns.

Modern structures frequently boast increased heights and spans necessitated by engineering requirements, demanding structures with ample strength and seismic resilience [**1**]. In the realm of civil engineering construction and application, various irregular cross-sectional steel tube concretes, notably those with round-end elliptical cross-sections, find widespread use [**2**]. The round-ended concrete-filled steel tube column (RE-CFST column) has gained recognition for its elevated load-bearing capacity, user-friendly construction process, aesthetically pleasing design and adaptable section layout [**3, 4**] (depicted in **Fig 1**).

Given the practical benefits offered by RE-CFST pipes, there is a growing trend towards conducting comprehensive research on their applications. For instance, Ding et al. [**5**] conducted a study on the mechanical performance of RE-CFST columns under static and quasi-static conditions. Their research employed a combination of experimental, finite element and theoretical methods. Additionally, they put forth pertinent calculation and design methods based on their findings. Zhang et al. [**6**] introduced a fiber beam column model to simulate the nonlinear performance of RE-CFST columns, a model that underwent validation through numerous examples. In a separate study, Wang et al. [**7**] performed a numerical analysis on eccentric compression in RE-CFST columns. Their analysis delved into the impacts of parameters such as diameter-to-thickness ratio, load eccentricity ratio and section slenderness ratio. Additionally, they proposed a simplified empirical formula for the eccentric bearing capacity of CFRE steel tube columns. Collectively, these investigations consistently highlight the advantageous characteristics of round-end sections, which exhibit a blend of constraints from both rectangular and circular sections, rendering them highly versatile.

Nonetheless, the frequent utilization of RE-CFST columns in bridge piers exposes them to harsh environmental conditions, posing a significant challenge to their long-term durability. A viable solution to address this challenge involves replacing the steel tube with corrosion-resistant aluminum alloy tubes. Aluminum alloy materials exhibit commendable corrosion resistance, a high strength-to-weight ratio, and ease of processing, rendering them suitable for deployment in corrosive environments or specialized structural components [**8**]. Bu et al. [**9**] once conducted axial compression performance tests and numerical studies on four circular end aluminum alloy tube concrete columns. The findings indicated that a large aspect ratio was the crucial factor influencing the axial compression performance of the columns. Building upon this insight, they proposed a simplified model and calculation method utilizing circular end aluminum tubes to effectively restrain concrete. However, it’s worth noting that ordinary aluminum alloy materials possess a relatively low elastic modulus (roughly one-third of that of ordinary steel), leading to decreased stiffness in aluminum alloy structures and heightened vulnerability to buckling failure of components. This observation aligns with the findings reported by Bu and his colleagues.

To enhance the mechanical performance of aluminum alloy structural components, researchers have proposed various forms of composite structural components, such as aluminum alloy tube concrete composites, carbon fiber reinforced plastic (CFRP) reinforced aluminum alloy composites and CFRP-aluminum alloy composite tube concrete composites, and have conducted fundamental studies on their mechanical performance. Khalil et al. [**10–13**] has proven that CFRP has good reinforcement effects and can significantly improve the impact resistance and stiffness of components. Regarding aluminum alloy tube concrete composites, Zhou and Young [**14, 15**] conducted axial compression performance tests on aluminum alloy reinforced concrete columns and studied the effects of different cross-sectional areas (square, rectangular and circular) and dimensions on their mechanical properties. Similarly, Zhou and Young [**16**] and Wang et al. [17] used a refined finite element model to analyze the axial compression performance of circular aluminum alloy tube concrete components and proposed a method for calculating bearing capacity. The research results on CFRP reinforced aluminum alloy composite components indicate that the main failure mode of these components is instability, with aluminum alloy tube playing a relatively limited role [**18**]. However, the CFRP aluminum alloy composite tube concrete composite component can significantly improve the stability and bearing capacity of aluminum alloy tubes. Zhu et al. [**19**] conducted bending performance tests and numerical simulations on square and rectangular CFRP aluminum alloy composite tube concrete components. Their results demonstrated that components wrapped with CFRP on all four sides exhibited higher bending stiffness and bearing capacity, and CFRP effectively delayed the occurrence of convex buckling of aluminum alloy tubes. Chen et al. [**20**] proposed strengthening aluminum alloy tube ocean concrete columns with CFRP, and proved through axial compression tests and FEA that CFRP can effectively alleviate the buckling of aluminum alloy tubes. They also proposed a calculation method for the axial compression bearing capacity of this composite column. The study revealed that CFRP can enhance the bearing capacity of aluminum alloy tube marine concrete and effectively suppress damage development.

**Fig 2:**
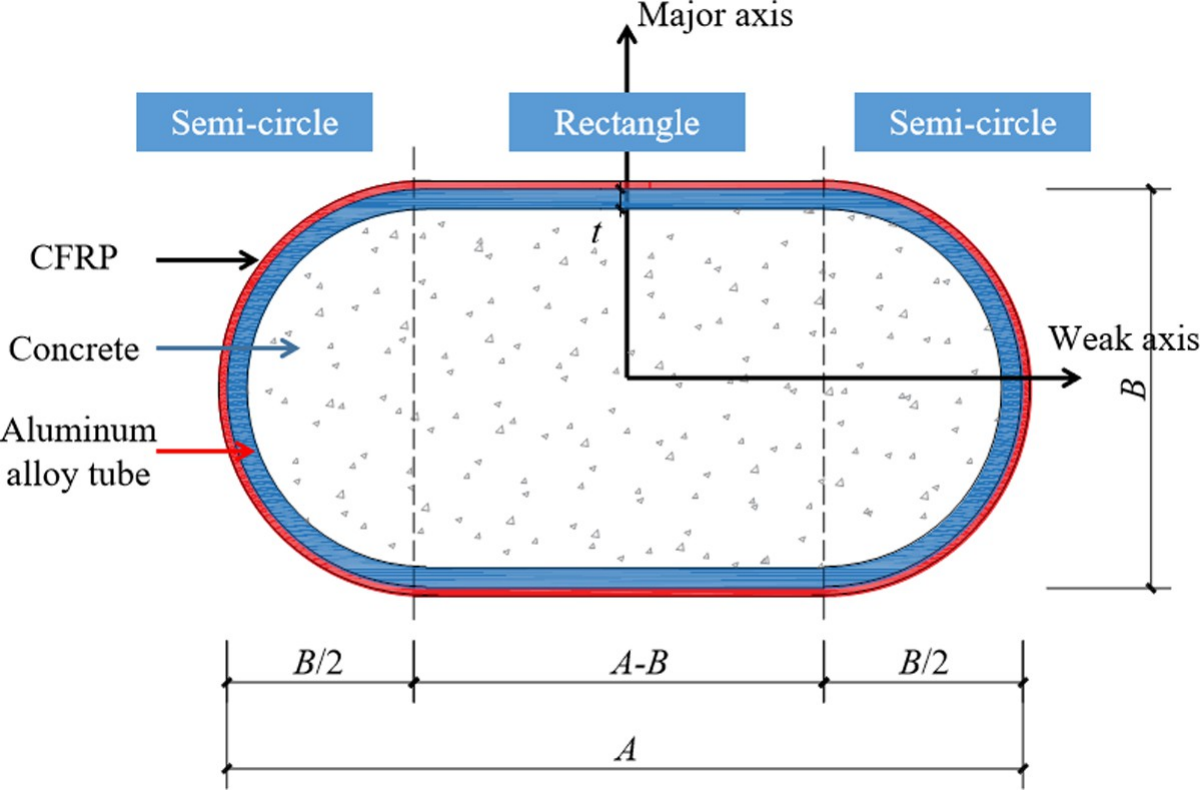
Construction of round-ended section.

On this basis, this study proposes a new type of hybrid column comprising oval aluminum tubes externally wrapped with CFRP and internally filled with concrete, as illustrated in **Fig 2**. Eight specimens were fabricated and tested under axial compression with the number of CFRP layers and aspect ratio being the varying parameters. The purpose is to provide a reference for the performance optimization and application of this structural form. In addition, ABAQUS, as an efficient finite element analysis software, is often used in nonlinear analysis of structures [**21**]. Therefore, this article also conducts finite element analysis and parameter analysis, which helps to analyze the sensitivity of influencing factors.

## Experimental program

### Design of specimen

A total of eight round-ended concrete-filled CFRP-aluminum tube column (RCFCAT) specimens were designed based on the number of CFRP layers and section aspect ratio. The specimens had a height (*H*) of 500 mm. Four section sizes were selected, as detailed in **Table 1**, with the section structure shown in **Fig 2**. In **Table 1**, *A* and *B* refer to the length and width of the aluminum alloy tube, respectively, *t* is the tube thickness, and *λ* is the slenderness ratio of the cross-section around the weak axis. The calculation method is as follows:

$$\lambda =\frac{H}{\sqrt{I/A_{\text{ac}}}}$$ (1)

where *H* is the height of the specimen. *I* is the second polar moment of area of the section, and *A*_ac_ is the area of the section.

**Table 1:** Design parameters of specimens.

Specimen *No*.	Height of specimen *H* /mm	Number of CFRP layers	*A×B×t* /mm	*A/B*	*λ*
CA100-0	500	0	100×50×2.5	2.0	36.7
CA115-0		0	115×45×5.0	2.5	40.2
CA120-0		0	120×30×2.0	4.0	59.3
CA130-0		0	130×65×2.0	2.0	28.2
CA100-1		1	100×50×2.5	2.0	36.7
CA115-1		1	115×45×5.0	2.5	40.2
CA120-1		1	120×30×2.0	4.0	59.3
CA130-1		1	130×65×2.0	2.0	28.2

Note: *A*, *B* represents the length and width of the cross-section; *t* is the thickness of the aluminum alloy tube; *λ* is the slenderness ratio of the specimen.

The round-ended section consists of symmetrical semicircles on left and right sides, and a rectangle in the middle. The specimen width (*B*) equals the semicircle radius. Specimens were produced through the following process: (1) Epoxy resin was applied evenly on the CFRP cloth surface before bonding it to the aluminum alloy tube. The CFRP tensile direction was circumferential to the tube, with an overlap length of *b* to prevent adhesive failure in the overlap region. (2) The CFRP-aluminum alloy tubes were air-dried in a cool place for 5 days before concrete pouring. Vibrating rods compacted the concrete during pouring to ensure quality. (3) After concrete initial set, both specimen ends were polished using a polishing machine to ensure flatness and avoid significant eccentricity during loading.

### Raw materials

C35 concrete was used to fabricate three standard cubic samples cured under same conditions as the RCFCAT columns. The mix proportion is shown in **Table 2**. Concrete cubic compressive strength (*f*_cu_) was tested as 36.8MPa one day before column testing. Single CFRP layer thickness was 0.167 mm with 500 mm width. Per manufacturer data, CFRP tensile strength and ultimate tensile strain were 3350 MPa and 0.0153, respectively. Aluminum alloy properties were obtained by tensile tests per GB/T 228.1–2010. Since the alloy lacked a clear yield point, the 0.2% residual strain strength (*f*_0.2_) was taken as the conditional yield strength based on references. Mechanical properties are listed in **Table 3**.

**Table 2:** Mix proportions of concrete (kg/m^3^).

Cement	Sand	Gravel	Water	Water-cement ratio
565	718	1076	232	0.41

**Table 3:** Material properties of aluminum tube.

Tube	*A* /mm	*B* /mm	*t* /mm	*f*_0.2_ /MPa	*f*_u_ /MPa	Elongation δ /%	Elastic modulus *E*_0_ /MPa
100×50×2.5	100	50	2.5	195.2	217.4	10.31	67500
115×45×5.0	115	45	5.0	199.7	209.2	13.22	66200
120×30×2.0	120	30	2.0	201.4	211.5	11.21	67100
130×65×2.0	130	65	2.0	197.3	214.1	12.43	68200

### Experimental apparatus and test procedure

**Fig 3:**
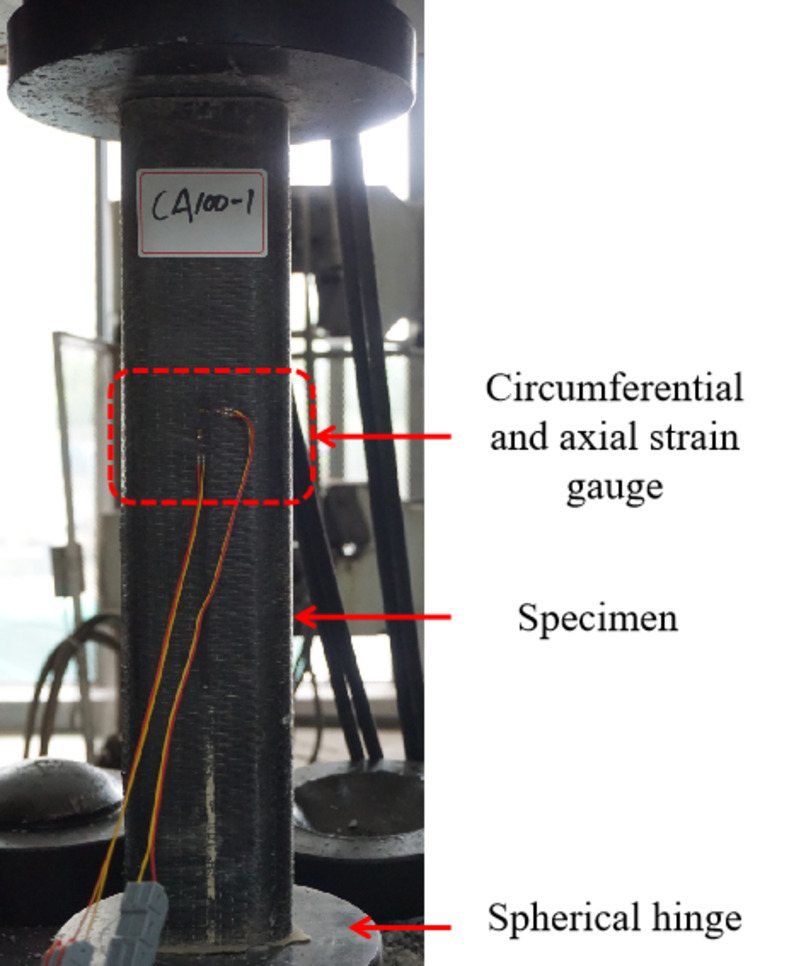
Loading device and strain gauge arrangement.

The loading device and measuring point arrangement for axial compression testing are shown in **Fig 3**. A YAW-1000 electro-hydraulic servo press applied axial load up to 100t maximum. Displacement control was used with 2mm/min loading rate. Two strain gauges (circumferential and axial) at mid-height monitored specimen and aluminum tube deformation.

## Result analysis

### Failure mode

**Fig 4:**
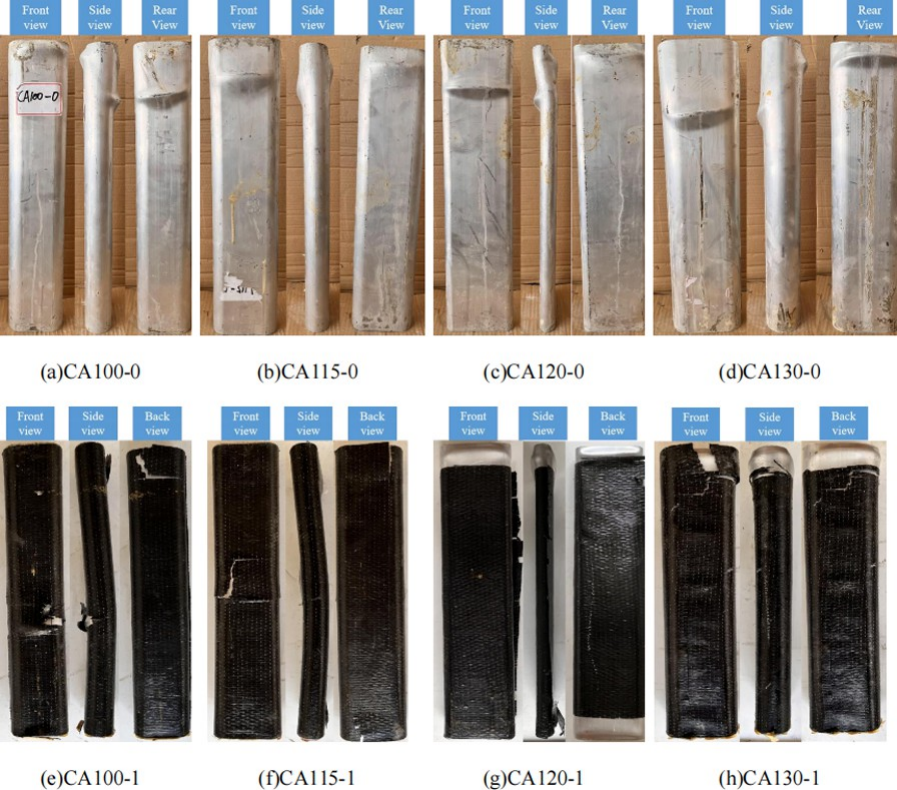
Failure mode.

**Fig 5:**
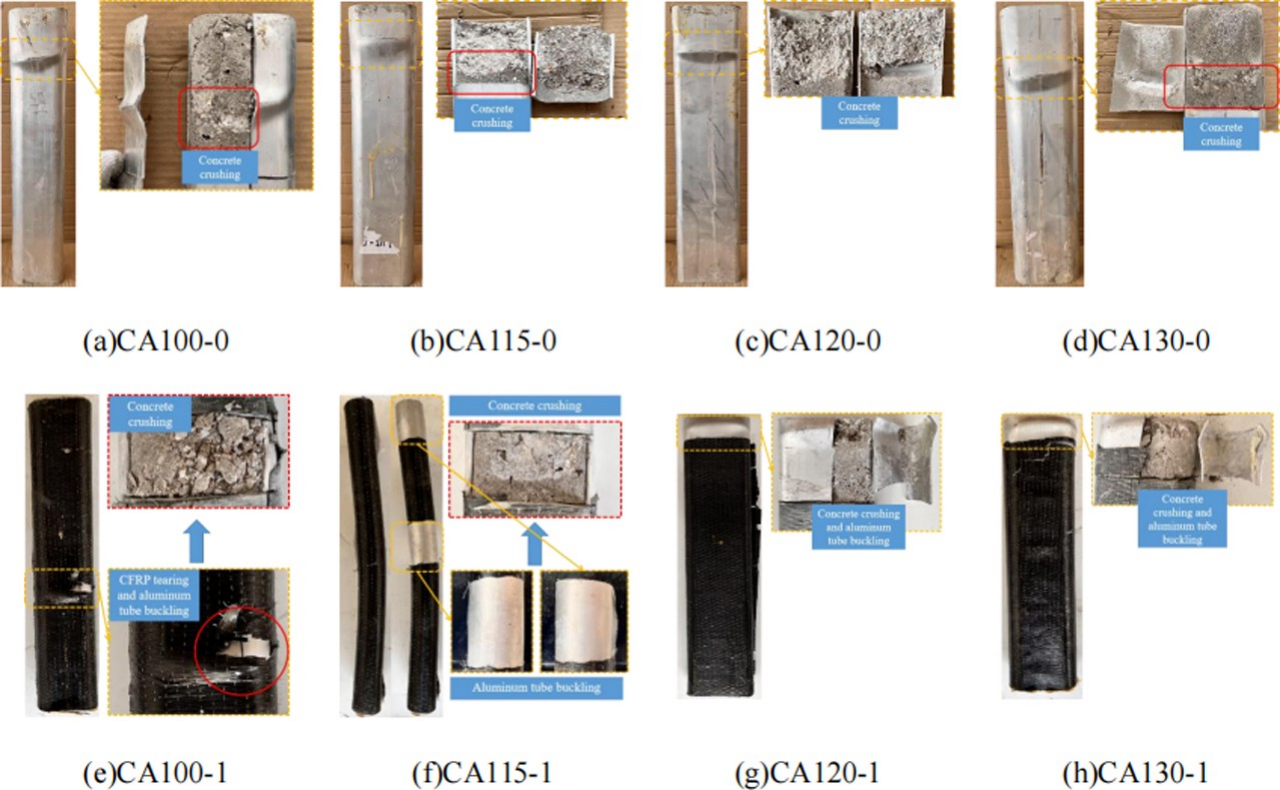
Internal failure.

The axial compression failure modes of all specimens are shown in **Fig 4**. For further investigation, the bulged aluminum tubes were cut open post-testing (**Fig 5**). The failure process was similar across specimens. At initial loading, no obvious change occurred as the aluminum tube and concrete core deformed together. With increased axial displacement, local buckling sequentially occurred on the two flat aluminum tube sides. For CFRP-wrapped specimens, tearing occurred at buckled positions.

From the final failure modes, most non-CFRP specimens underwent local buckling failure dominated by shear-slant deformation. The difference is that the CA100-0, CA115-0 and CA130-0 specimens experienced oblique shear failure caused by local bulging, while the CA120-0 specimen not only experienced local bulging, but also experienced overall instability failure, causing the concrete to be sheared off. For specimen CA120-0 with a large aspect ratio, the significant difference between major and minor axis moments of inertia resulted in relatively thin concrete on the minor axis side. Thus, in addition to local buckling, overall instability failure occurred, leading to concrete core shear failure. CFRP-wrapped specimens initially failed by overall flexural bending followed by local buckling at large deformation, during which CFRP tearing occurred. This indicates the CFRP constrained aluminum tube lateral deformation during loading. After cutting open, it was noticed the concrete was crushed and filled the bulged tubes at buckled positions. This occurred because the flat sides provide weaker confinement than the curved ends, leading to concrete core compression failure.

### Axial load-displacement curve

**Fig 6:**
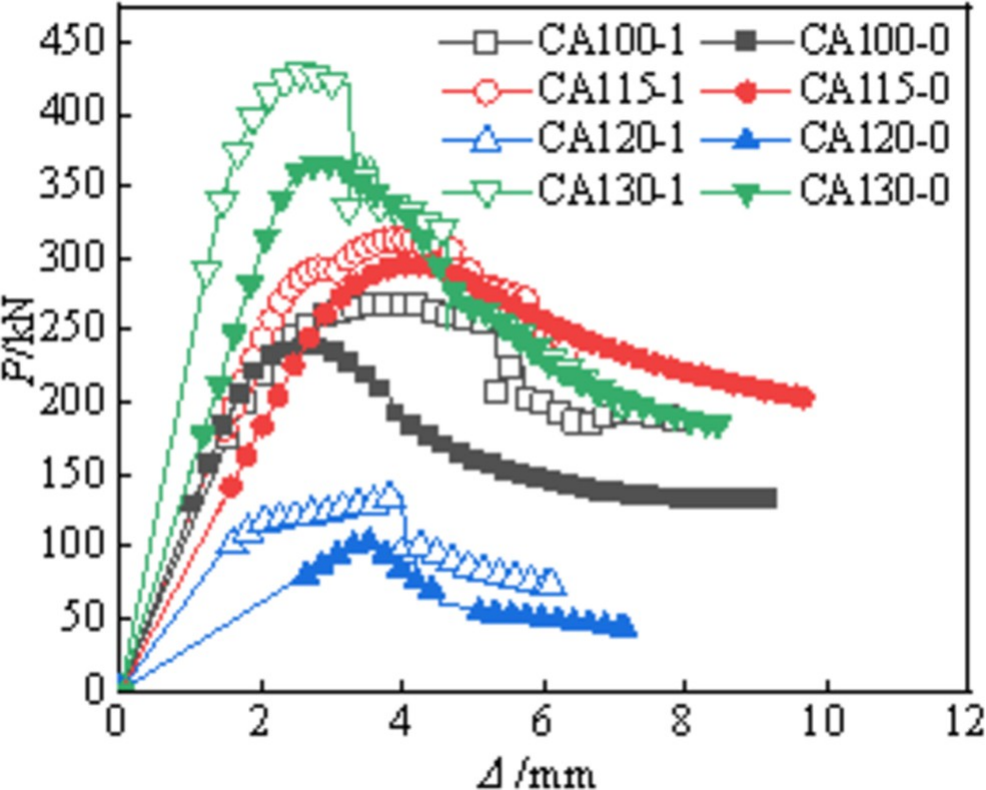
Load-axial displacement curve.

**Fig 6** shows the axial load-displacement curves (*P*-*Δ*) of all specimens (S1 Data). The *P*-*Δ* of each specimen exhibits a similar shape in the figure. It shows an elastic stage before reaching a peak load of approximately 0.6 times. Afterward, the aluminum alloy tube undergoes plastic deformation, entering the plastic stage. For specimens reinforced with CFRP, when CFRP reaches its ultimate strength, the specimen simultaneously reaches its ultimate bearing capacity. At the moment of CFRP fracture, there is a sudden drop in the curve. On the other hand, specimens without CFRP reach their extreme point when the core concrete reaches its ultimate strength. Then, the aluminum alloy tube exerts a restraining effect, causing the curve to slowly decrease. Strengthening with CFRP significantly improves the axial stiffness and bearing capacity of the circular end aluminum alloy tube concrete column. Additionally, the slope of the descending section of the curve decreases significantly, indicating that CFRP also enhances the axial deformation resistance of the specimen.

### Bearing capacity and ductility

**Fig 7:**
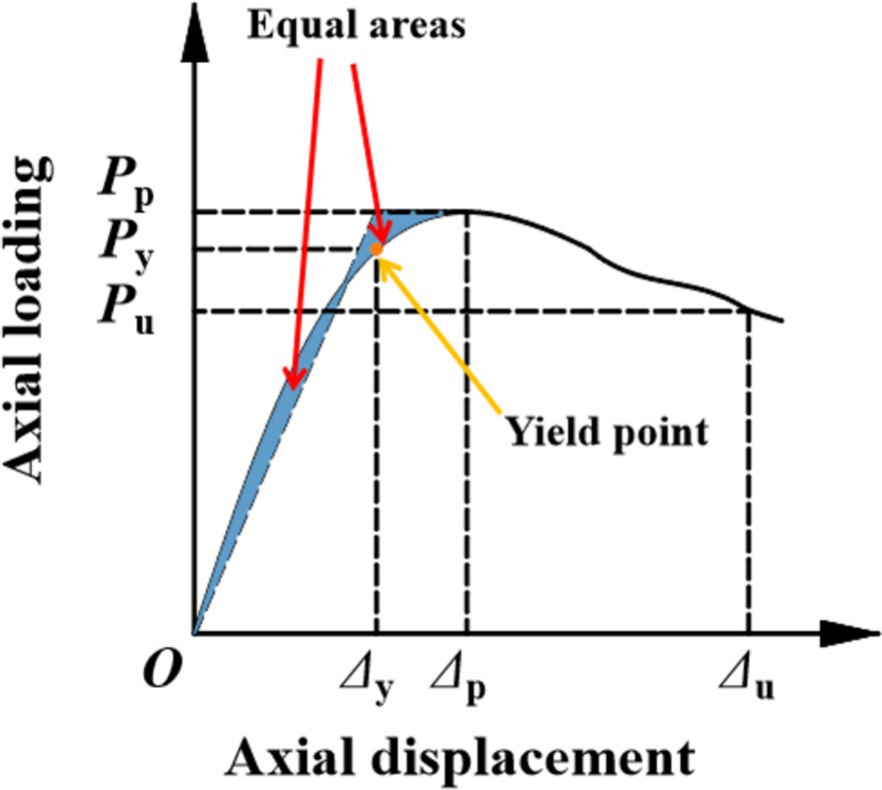
Calculation model for ductility coefficient.

**Table 4** presents the key mechanical performance indicators of all specimens. The ductility coefficient *μ* is calculated using the area equivalence method [**22**], as shown in **Fig 7**. Ductility can be assessed by the ductility ratio *μ* calculated by

$$\mu =\frac{\Delta_{\text{u}}}{\Delta_{\text{y}}}$$ (2)

Where *Δ*_u_ was the axial displacement when the load decreases to 85% of the peak load.

**Table 4:** Test results.

Specimen *No*.	Initial stiffness *K* /kN∙mm^-1^	Ultimate load *P*_u_ /kN	Theo. ultimate load *P*_s_ /kN	*SI*	*μ*	FE result *P*_FE_ /kN	*P*_u_/*P*_FE_
CA100-0	125.2	238.4	228.0	1.05	1.95	249.7	0.95
CA100-1	129.0	269.2	228.0	1.18	2.12	269.8	1.00
CA115-0	90.4	295.4	345.3	0.86	1.88	305.0	0.97
CA115-1	124.7	313.5	345.3	0.91	2.14	311.9	1.01
CA120-0	30.7	103.2	183.0	0.56	1.15	100.3	1.03
CA120-1	66.9	135.0	183.0	0.74	1.65	145.6	0.93
CA130-0	153.4	365.6	323.7	1.13	1.79	367.7	0.99
CA130-1	240.1	428.6	323.7	1.32	1.85	432.8	0.99
Average							0.983
Standard deviation							0.032
Coefficient of variation							0.032

For comparison and analysis, the theoretical ultimate load (*P*_s_) without confinement effect was calculated using the superposition method. The load capacity coefficient (*SI*) was obtained by dividing the experimental value by *P*_s_. When *SI* is greater than 1, the combined strength of aluminum alloy tubes and concrete is greater than the strength of the material itself, indicating that aluminum alloy tubes provide constraints on the core concrete. The calculation method is as follows:

$$SI=\frac{P_{\text{u}}}{P_{\text{s}}}$$ (3)

$$P_{\text{s}}=A_{\text{a}}f_{0.2}+A_{\text{c}}f_{\text{c}}$$ (4)

where, *P*_s_ and *P*_u_ are the theoretical calculated ultimate bearing capacity and the experimental measured ultimate bearing capacity, respectively. *A*_a_ and *A*_c_ are the cross-sectional areas of aluminum alloy tubes and concrete, respectively. *f*_0.2_ and *f*_c_ are the yield strength and compressive strength of aluminum alloy tubes and concrete, respectively.

It can be seen from the **Table 4** that the axial load carrying capacity and ductility of the specimens are significantly improved after CFRP strengthening of the aluminum alloy tubes. With the increase of aspect ratio *a/b*, the effectiveness of CFRP in enhancing the axial capacity and ductility increases to some extent. Specifically, the increase in axial load capacity ranges from 6.1% to 30.8%, and the increase in ductility ranges from 3.3% to 43.4%. This is similar to the research results of Chen et al. [**20**], which may be because CFRP has the characteristics of high strength and high elastic modulus, which can effectively constrain the local yield of the tube wall. On the other hand, after the aluminum alloy tube is reinforced with CFRP, the concrete in the core area is subjected to stronger confinement, and according to the confinement concrete theory, the ductility of the concrete will also be improved. Therefore, the overall ductility has been improved.

For both series of specimens (with and without CFRP), as *a/b* increases, *SI* and ductility coefficient decrease gradually. When *a/b* = 2.5 and *a/b* = 4.0, the enhancement coefficients are less than 1, indicating degradation of the specimens. The critical aspect ratio is between 2.0–2.5. Beyond this range, the specimens have a relatively slender section, thus second-order effects become more significant, leading to overall instability. After CFRP strengthening, the degradation rates of *SI* and ductility coefficient are mitigated, demonstrating the confinement provided by CFRP and improved deformation capacity.

For specimens with *a/b* = 4 (CA120-0 and CA120-1), the increases in axial capacity and ductility are most noticeable, reaching 30.8% and 43.4% respectively. This is because the aspect ratio is the largest and premature overall instability occurred in CA120-0. The CFRP significantly enhanced the deformation capacity and delayed the instability failure. Therefore, the axial performance improvement of circular-ended aluminum alloy concrete columns by CFRP is most pronounced for this slenderness ratio.

### Stiffness degradation

**Fig 8:**
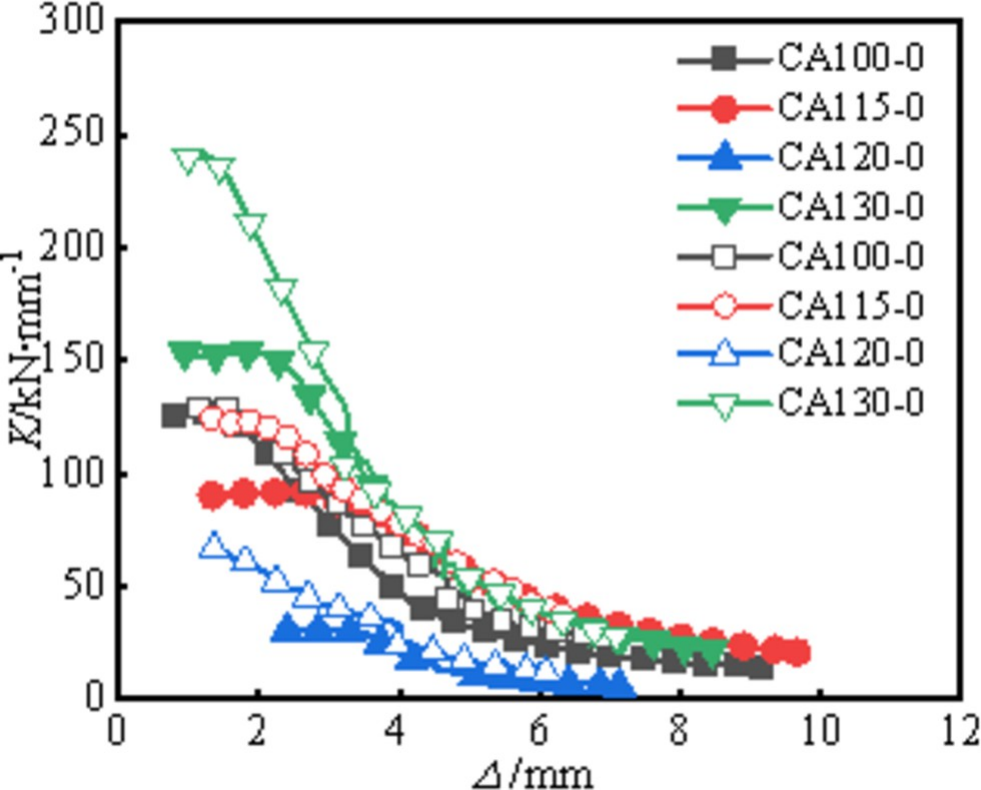
Stiffness degradation curve.

The stiffness degradation curves of each specimen are shown in **Fig 8** and the initial axial compression stiffness of each specimen are shown in **Table 4**. It can be observed from the figures and tables that the stiffness degradation curves of all specimens have similar shapes. The degradation rate is fastest when the axial displacement is greater than about 2mm. The stiffness degradation curves tend to plateau when the axial displacement reaches around 4mm. This is because after the specimens enter the elastic-plastic stage, the aluminum alloy tube deforms rapidly, leading to a quick reduction in section stiffness. This is because after the elastic stage of the specimen, the material enters the plastic stage, and at this time, the bearing capacity and deformation are non-linear, resulting in a decrease in stiffness. The difference is that the initial axial stiffness of the circular-ended aluminum alloy concrete columns is significantly improved after CFRP strengthening, with increases ranging from 3.0% to 117.9%. This is attributed to the high elastic modulus and strength of CFRP, which enhances the constraint on the lateral expansion deformation of the concrete core and aluminum tube, delays local buckling deformation of the aluminum tube, and thus improves the overall stiffness. This demonstrates that CFRP can effectively increase the axial stiffness of this type of composite columns. As *a/b* increases, the initial axial stiffness decreases gradually for all specimens, because the slender ratio along the weak axis increases, causing premature overall instability and lower initial axial stiffness. The axial stiffness improvement is most significant when *a/b* = 4 (CA120-0 and CA120-1), since the specimens have the largest aspect ratio. Before CFRP rupture, bending of specimens has initiated, and the high elastic modulus of CFRP restrains the bending, leading to the most noticeable increase in axial stiffness.

### Strain analysis

**Fig 9:**
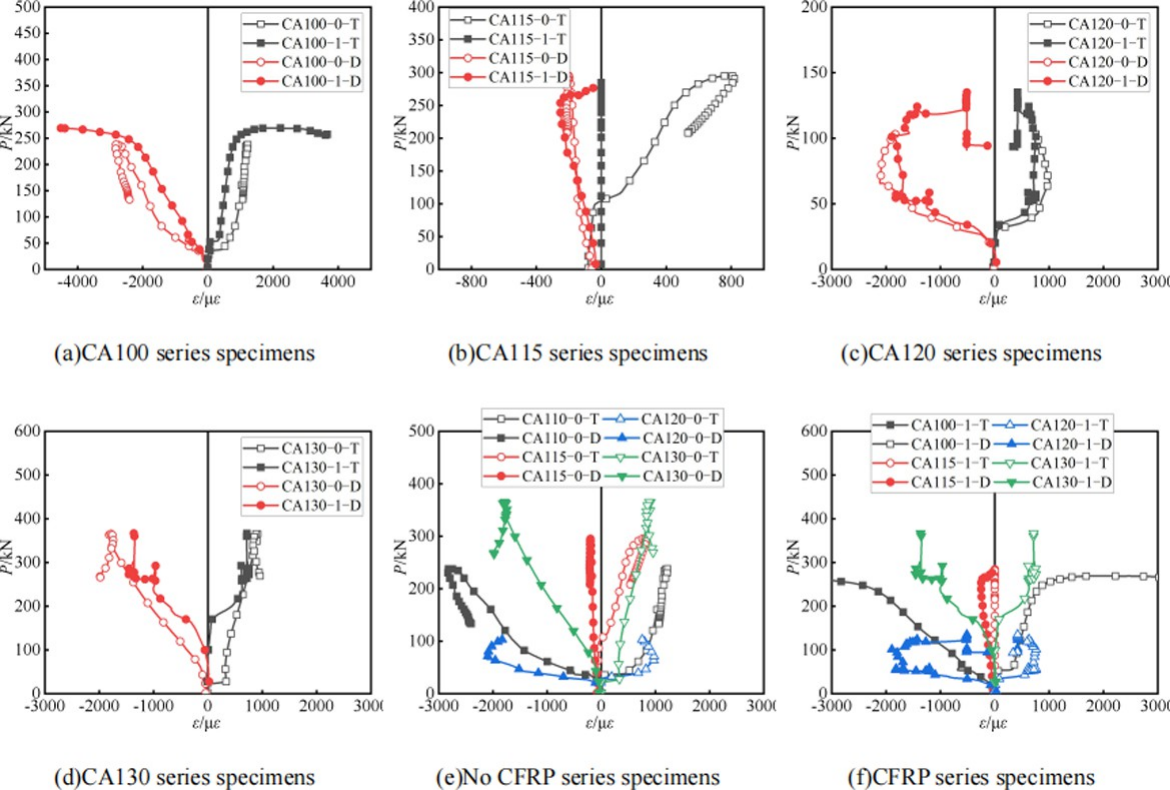
Load-strain curve.

**Fig 9** shows the strain development of the specimen, T represents circumferential strain, D represents axial strain. The figure shows that in the early loading stage, the circumferential and axial strains develop concurrently, indicating an elastic stage with minimal lateral constraint. Upon entering the plastic stage, the circumferential strain increases at a faster rate due to confinement from the aluminum tube. Under the same load level, the axial strain on the surface of the aluminum alloy tube is significantly reduced after CFRP bonding, indicating that the CFRP layer can effectively confine and share the concrete compression deformation, thus reducing the strain in the tube wall. The good bond between CFRP and aluminum alloy tube ensures their synergetic works, with part of concrete compression deformation absorbed by CFRP. The high stiffness of CFRP also provides confinement to the tube wall, validating its potential in enhancing the load capacity of aluminum-concrete composite sections.For the control groups with different aspect ratios, the strains in both orientations are evenly distributed and relatively larger under the same load when *a/b* is around 2.0 (CA100 series), indicating the best confinement effect of the round-ended aluminum tube, which is close to the uniform compression deformation condition of a circular section.

## Finite element analysis (FEA)

### Materials

#### Aluminum

Previous studies have shown that the constitutive curve of aluminum alloy is significantly different from that of ordinary carbon steel because there is no obvious yield step in its Stress–strain curve. Based on previous research, Chen et al. [**20**] selected the constitutive model for simulating aluminum alloy tube confined marine concrete columns. This model was proposed by Ramberg-Osgood [23] and has been widely used to simulate the compressive response of aluminum alloys. This model can effectively reproduce the different nonlinear behaviors of aluminum alloy and steel. The constitutive law can be expressed as follows:

$$\varepsilon =\left\{\begin{array}{l} \frac{f}{E_{0}}+0.002{\left(\frac{f}{f_{0.2}}\right)}^{n}\;f\leq f_{0.2}\;\\ \frac{\left(f-f_{0.2}\right)}{E_{0.2}}+{\left\{\frac{0.008-\left(f_{1.0}-f_{0.2}\right)}{E_{0.2}\frac{\left(f-f_{0.2}\right)}{\left(f_{1.0}-f_{0.2}\right)}}\right\}}^{n{'}_{0.2,1.0}}\;f\geq f_{0.2} \end{array}\right.$$ (5)

where the strain (*ε*) and stress (*f*) of aluminum are considered. The 0.2% proof stress (*f*_0.2_) and 1% proof stress (*f*_1.0_) are also taken into account. Additionally, the strain at *f*_0.2_ (*ε*_0.2_) and the elastic modulus of the aluminum alloy (*E*_0_) are considered. The stiffness at *f*_0.2_ is denoted as *E*_0.2_. The values for *n’*_0.2,1.0_ can be obtained from the research conducted by Wang et al. [17].

#### Concrete

There are two methods in existing literature for simulating constrained concrete columns. One method is to directly use the constitutive law of constrained concrete, taking into account the improvement of strength in the material properties of concrete. Another approach is to use a uniaxial constitutive model of concrete, which automatically considers constraints using software. Due to the consideration of the buckling of aluminum alloy tubes and the contact between concrete and aluminum alloy tubes in this article, a uniaxial stress model was selected. In ABAQUS software, the concrete damage plasticity model (CDP) was adopted. The constitutive equation of concrete specified in GB50010-2010 “Code for design of reinforced concrete structures” [**24**] is adopted in this article, and the calculation formula is as follows:

$$y=\left\{\begin{array}{l} \alpha_{\text{a}}x+\text{(}3-2\alpha_{\text{a}}\text{)}x^{2}+\text{(}\alpha_{\text{a}}-2\text{)}x^{3}\;0\leq x\leq 1\\ \;\frac{x}{\alpha_{\text{d}}{\text{(}x-1\text{)}}^{2}+x}\;x\geq 1 \end{array}\right.$$ (6)

$$y=\frac{\sigma }{f_{\text{c}}},\;x=\frac{\varepsilon }{\varepsilon_{\text{c}}}$$ (7)

where *f*_c_ and *ε*_c_ represents the ultimate compressive stress and strain of concrete; *α*_a_ and *α*_d_ represents the parameters of the ascending and descending segments, respectively.

#### CFRP

In ABAQUS, the progressive fiber damage model proposed by Hashin [**25**] is often used to describe the damage evolution of fiber materials. This model includes four independent damage modes with the following functions:

$$F_{f}^{t}={\left(\frac{\sigma_{11}}{X^{T}}\right)}^{2}+\alpha {\left(\frac{\tau_{21}}{S^{L}}\right)}^{2}-1\leq 0,\;\sigma_{11}\geq 0$$ (8)

$$F_{f}^{\text{c}}={\left(\frac{\sigma_{11}}{X^{C}}\right)}^{2}-1\leq 0,\;\sigma_{11}\leq 0$$ (9)

$$F_{m}^{t}={\left(\frac{\sigma_{22}}{Y^{T}}\right)}^{2}+{\left(\frac{\tau_{12}}{S^{L}}\right)}^{2}-1\leq 0,\;\sigma_{22}\geq 0$$ (10)

$$F_{m}^{\text{c}}={\left(\frac{\sigma_{22}}{2S^{L}}\right)}^{2}+\left[{\left(\frac{Y^{C}}{2S^{L}}\right)}^{2}-1\right]\frac{\sigma_{22}}{Y^{C}}+{\left(\frac{\tau_{12}}{S^{L}}\right)}^{2}\;-1\leq 0,\;\sigma_{22}\leq 0$$ (11)

where *σ*_11_, *σ*_22_, *τ*_12_ were the stress component; *X*^T^, *X*^C^ were the tensile strength and compressive strength along the fiber length; *Y*^T^, *Y*^C^ were the tensile strength and compressive strength of the fiber in the vertical direction; *S*^L^ was the shear strength. The damage parameters used in this article are shown in **Table 5**.

**Table 5:** CFRP material properties [26].

Elastic (Gpa)						Hashin’s damage Model (Mpa)					
*E*_1_	*E*_2_	*υ*_12_	*G*_12_	*G*_13_	*G*_23_	*X* ^*T*^	*X* ^*C*^	*Y* ^*T*^	*Y* ^*C*^	*S*_*12*_	*S*_*13*_
211.49	7.93	0.35	5.3	5.3	4	3300	1414	134	169	134	120

### Mesh

**Fig 10:**
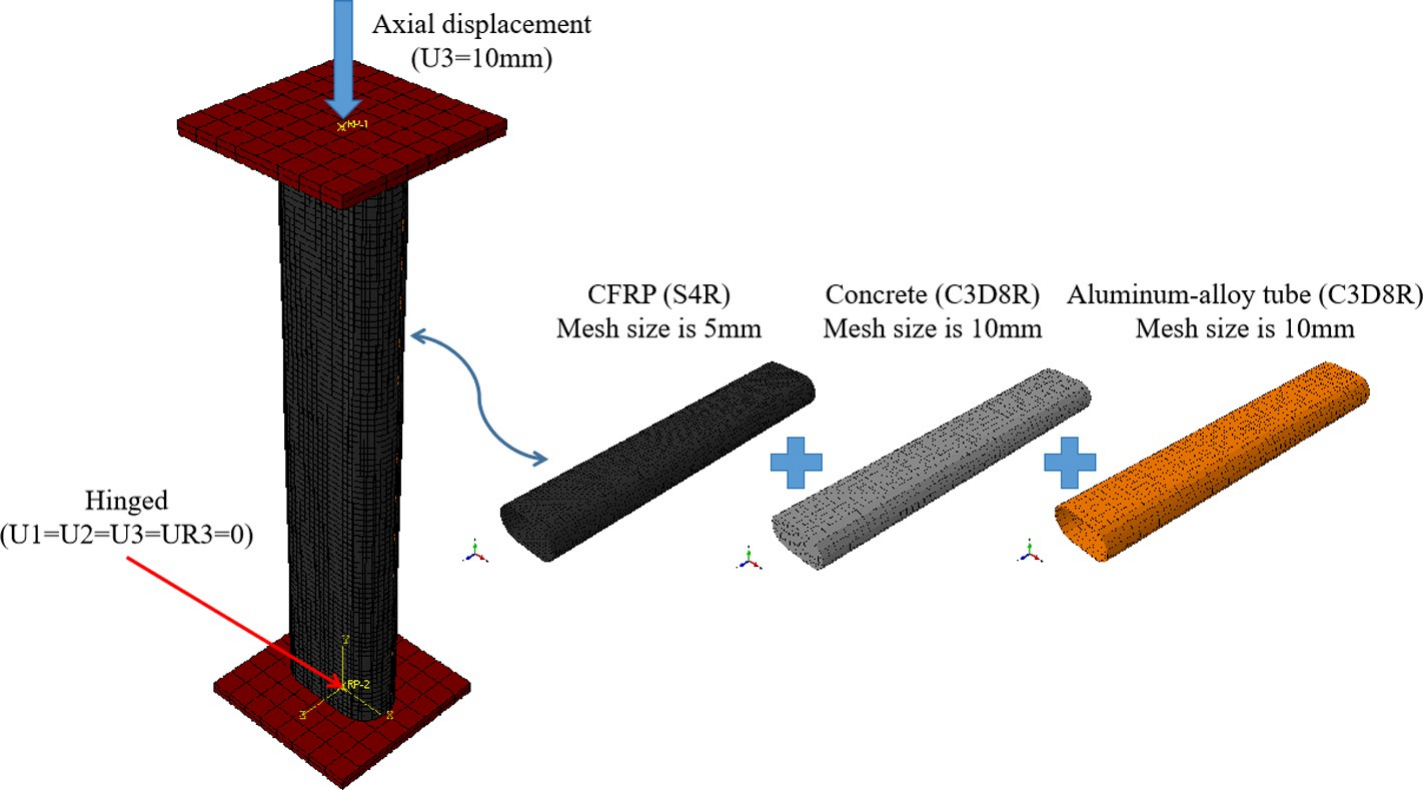
Model production.

**Fig 11:**
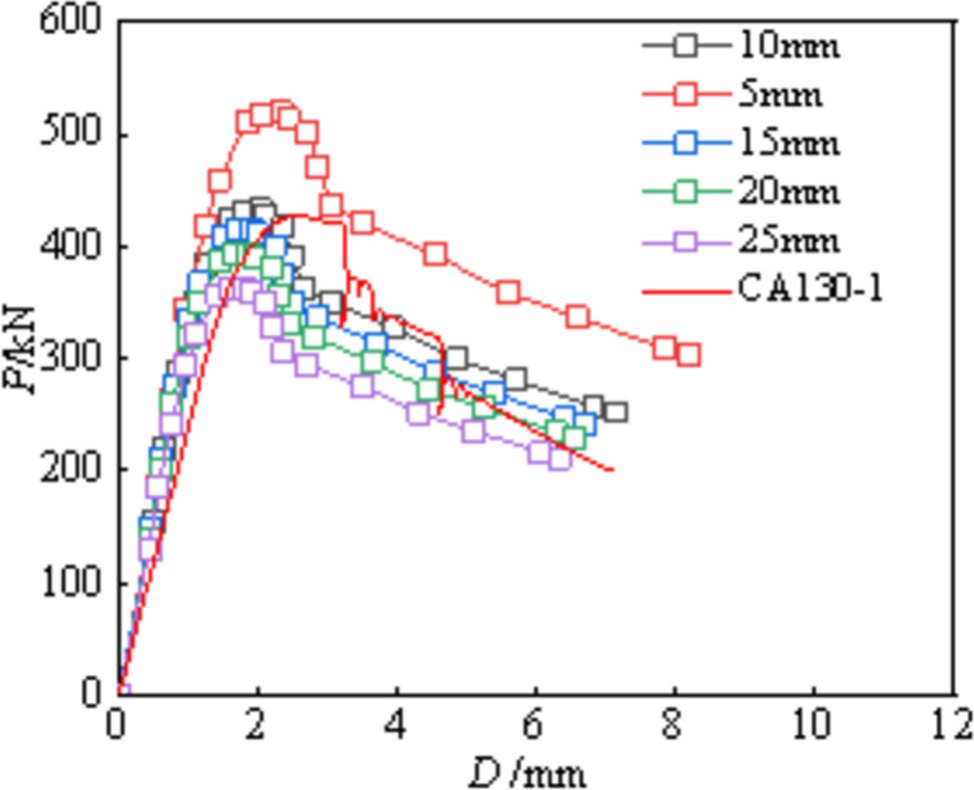
Mesh sensitivity analysis.

The specimen model used C3D8R solid elements for the concrete and aluminum alloy tubes, and S4R shell elements for the CFRP to better capture rupture behavior (**Fig 10**). Rigid loading pads were modeled at the top and bottom to facilitate load and boundary condition applications. The selection of mesh size has undergone multiple experiments and calculations, as shown in **Fig 11**. As shown in the figure, the calculation results vary with different grid sizes. In order to balance calculation time and accuracy, 10 mm mesh are used for concrete and aluminum alloy tube, while 5 mm mesh are used for CFRP [**20**].

### Construction of the model

The interaction between the concrete and aluminum alloy tube was simulated using a surface-to-surface contact approach. In the tangential direction, a penalty function with a friction coefficient of 0.5 was used to define the contact between the concrete and aluminum tube [20]. In the normal direction, a hard contact model was employed. To model the hard debonding behavior observed experimentally, the aluminum tube and CFRP were tied together in the simulation.

Boundary conditions were applied to simulate the spherical hinge used in the test. At the bottom of the specimen, displacements in three directions and rotation along the axis were constrained. At the top loading end, a 10mm axial displacement matching the test was applied without constraining displacements and rotations in other directions.

### Model validation

**Fig 12:**
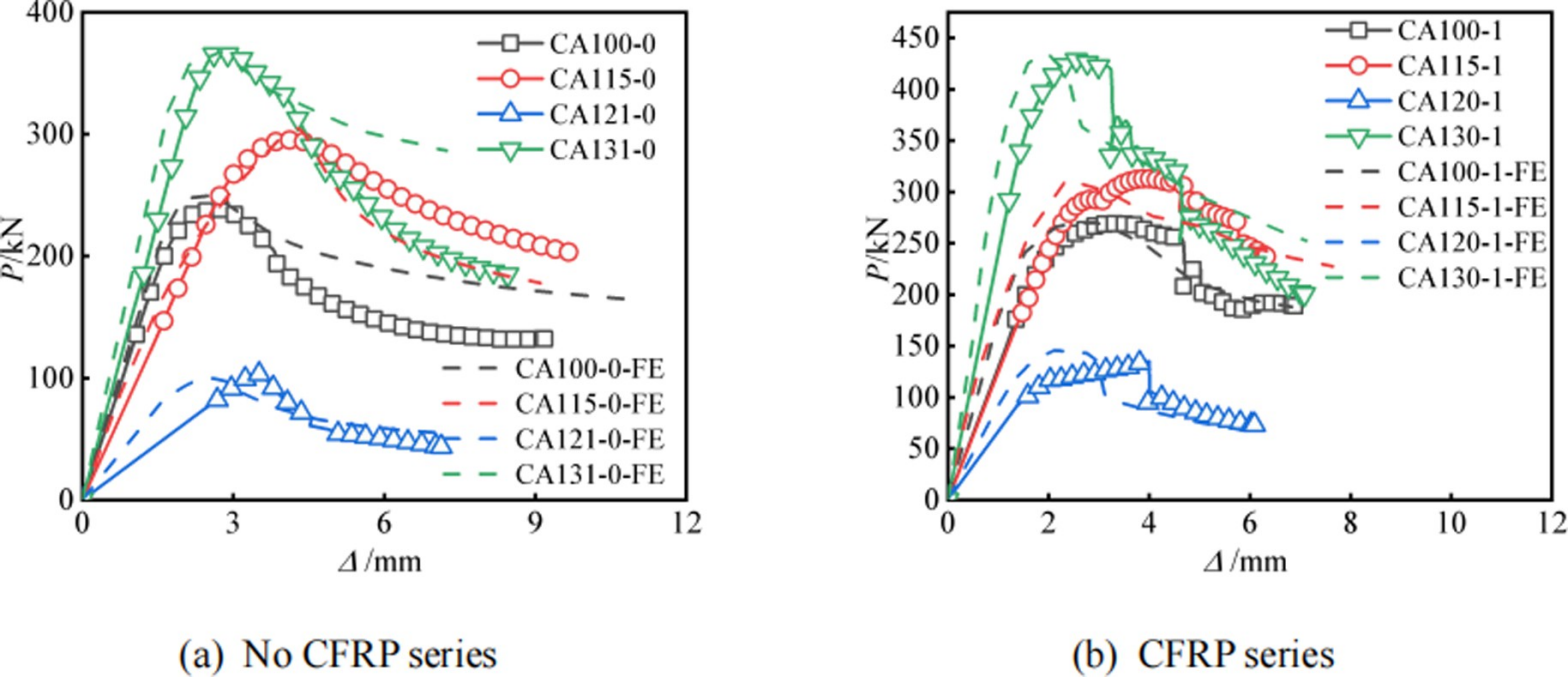
Comparison of axial load-displacement curve.

**Fig 12** shows a comparison of the axial load-displacement curves of all specimens. From the figure, it can be seen that the axial load displacement curve simulated by the finite element method is highly consistent with the experimental results, and the errors mainly exist in the initial stiffness and ultimate load. The reason for this error is that there are initial gaps between the two ends of the specimen and the loading equipment before loading, and there are also gaps inside the concrete. Therefore, during the loading process, the voids are continuously compressed, and the axial displacement will also be too large. The finite element model cannot carefully consider this aspect. From the calculation results in Table 4, it can be seen that the average ratio of the ultimate load between the test and finite element simulation is 0.983, with an error of only 1.7%. And the coefficient of variation is 0.032, indicating stable results.

**Fig 13:**
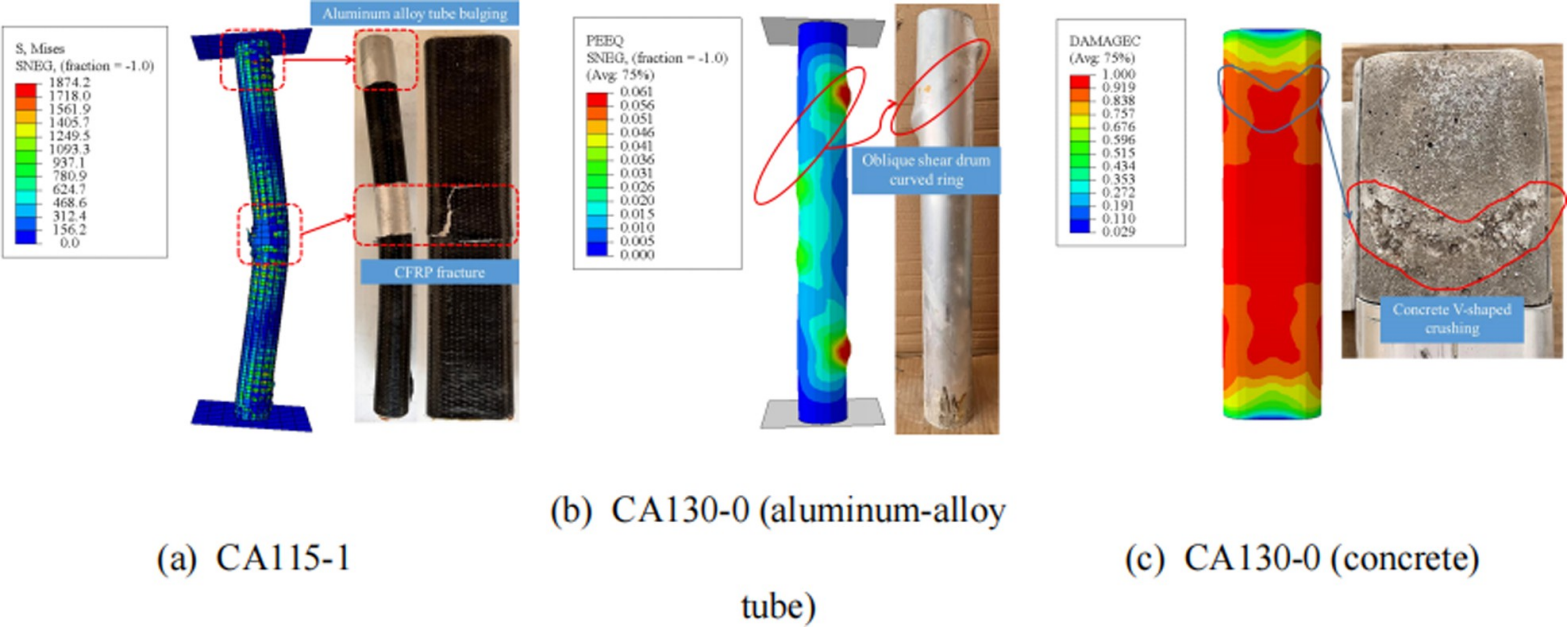
Comparison of failure modes.

A comparison of the failure modes between representative specimens and finite element models is shown in **Fig 13**. For CA115-1, it is evident from the calculation results of the model that there is tearing of the external CFRP, with protrusions in the middle and top of the aluminum tube outward. Moreover, for the specimen (CA130-0) without CFRP adhesive, it was observed that the internal concrete exhibited downward compression damage on both sides and bulging deformation of the aluminum tube. This is very close to the experimental results.

The above results indicate that the finite element model established in this article has been well validated and can provide reference for subsequent research.

### Parameter analysis

**Fig 14:**
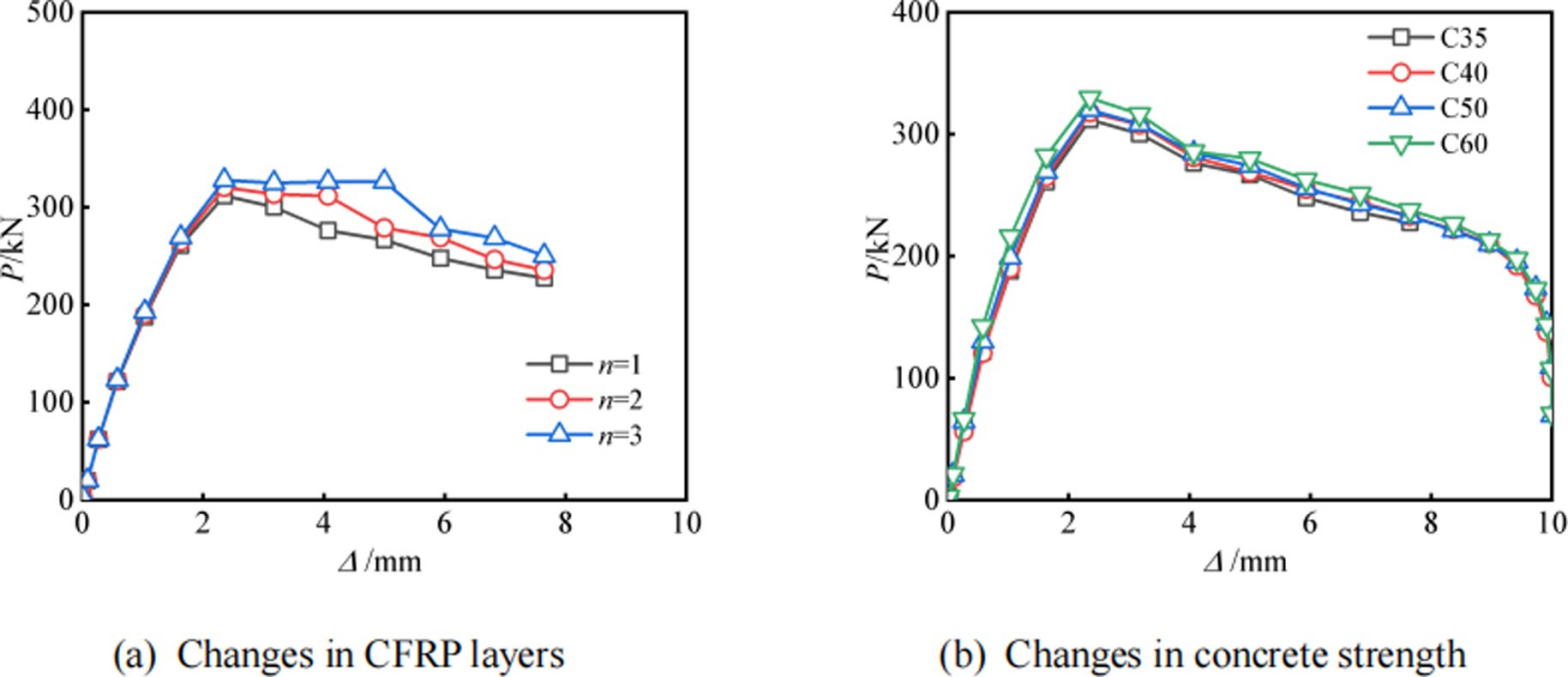
Load-displacement curve for parameter analysis.

The limited number of experimental samples indicates that more parametric analyses with additional samples are required to fully study RCFCAT performance. CA115-1 was selected as the benchmark model to study the effects of different CFRP layers and concrete strength. Because the CA115-1 model restores the bulging and CFRP fracture of aluminum alloy tubes, and has high consistency in the *P*-*Δ* curve. **Fig 14** presents the *P*-*Δ* curves for all specimens in the parametric analysis. As shown in the figure, with the increase of the number of CFRP layers, the ultimate bearing capacity of RCFCAT is significantly improved, and the displacement reaching the extreme point is significantly increased, indicating a significant improvement in its deformation resistance. Compared with the specimens with one layer of CFRP, the ultimate bearing capacity of the specimens with three layers of CFRP increased by 5.1%. Improving the strength of core concrete has little effect on the shape and ultimate bearing capacity of the curve. Therefore, increasing the number of CFRP layers is one of the most effective ways to improve the ultimate bearing capacity of RCFCAT. Overall, the number of CFRP layers can have a greater impact on the axial compression performance of specimens than the strength of concrete, and should be emphasized in subsequent research.

## Conclusions

1. The failure mode of CFRP aluminum alloy tube confined concrete columns is similar to that of CFRP steel tube concrete, and the failure mode is between rectangular and circular sections. All specimens exhibited similar sequential local buckling failures occurring on the two flat planes of the circular-ended aluminum tubes. For CFRP-strengthened specimens, CFRP rupture was observed at the buckled positions. Unstrengthened specimens failed primarily due to local buckling under oblique shear. Specimens with aspect ratios greater than 4 showed overall instability failures, initially demonstrating bending failures throughout the structure before subsequent local buckling, CFRP rupture, and failure.
2. The presence of CFRP improves the confinement of aluminum alloy pipes to the core concrete, thereby improving bearing capacity and ductility. For tubes with the same section dimensions, CFRP strengthening increased the maximum axial load-carrying capacity and ductility of the aluminum alloy tubes by up to 30.8% and 43.4%, respectively. As the section aspect ratio increased, the enhancement coefficients for load capacity and ductility decreased gradually. Confinement effect was optimal at aspect ratio around 2.0.
3. All specimens exhibited similar stiffness degradation curves. After entering the elasto-plastic stage, rapid deformation of the aluminum alloy tube led to a quick reduction in section stiffness. CFRP strengthening increased the initial axial stiffness by up to 117.9%. The initial axial stiffness decreased gradually as the aspect ratio increased, with the most significant axial stiffness enhancement occurring at an aspect ratio of 4.
4. Analysis of circumferential and longitudinal strains on the flat planes showed that CFRP bonding substantially decreased strains in both orientations compared to the same load without CFRP. The confinement effect provided by the circular-ended tubes was optimal at an aspect ratio of approximately 2.0.
5. The established ABAQUS FEA model has been validated, with the simulated axial load-displacement curve closely matching experimental results. The average error of the ultimate load ratio is only 1.7%. The model effectively reproduced the aluminum alloy tube bulging, CFRP fracture, and internal concrete V-shaped crushing observed experimentally.
6. The parametric analysis indicates increasing the CFRP layer number improves RCFCAT’ ultimate bearing capacity and deformation resistance, representing one of the most effective ways to increase RCFCAT’s ultimate bearing capacity. However, increasing the core concrete strength has little effect on the load-displacement curve shape or ultimate bearing capacity.
7. Overall, RCFCAT has good ductility and load-bearing capacity, but its application in structures still requires consideration of the construction of CFRP. This article only conducted monotonic load tests, and further research is needed on eccentric compression performance, shear performance, seismic performance, and other aspects.

## Supporting information

S1 Data (XLS)
